# Causal Beliefs and Perceptions of Risk for Diabetes and Cardiovascular Disease, the Netherlands, 2007

**Published:** 2011-10-15

**Authors:** Liesbeth Claassen, Lidewij Henneman, Danielle Timmermans, Giel Nijpels, Jacqueline Dekker, Theresa Marteau

**Affiliations:** Department of Public and Occupational Health, EMGO Institute for Health and Care Research, VU University Medical Center; EMGO Institute for Health and Care Research, VU University Medical Center, Amsterdam, the Netherlands; EMGO Institute for Health and Care Research, VU University Medical Center, Amsterdam, the Netherlands;; EMGO Institute for Health and Care Research, VU University Medical Center, Amsterdam, the Netherlands; EMGO Institute for Health and Care Research, VU University Medical Center, Amsterdam, the Netherlands; Health Psychology Section (at Guy’s), King’s College, London, United Kingdom

## Abstract

**Introduction:**

Understanding people's perceptions of disease risk and how these perceptions compare with actual risk models may improve the effectiveness of risk communication. This study examined perceived disease risk and causal beliefs for type 2 diabetes and cardiovascular disease (CVD), the relationship between self-reported risk factors and perceived disease risk, and the influence of causal beliefs on perceived disease risk in people at increased risk.

**Methods:**

The sample (n = 255) consisted of people who were at increased risk for diabetes and CVD (aged 57-79 y). Participants completed a postal questionnaire assessing risk factors, perceived risk, and causal beliefs for diabetes and CVD. We used regression analyses to examine the relationship between risk factors and perceived disease risk and to explore how causal beliefs affect the relationship between risk factors and perceived disease risk.

**Results:**

Associations between risk factors and perceived diabetes and CVD risks were weak. Perceived risk, causal beliefs, and explained variance of risk factors on perceived risk were lower for diabetes than for CVD. Stronger beliefs concerning 1) overweight as a cause of diabetes and 2) smoking as a cause of CVD strengthened the association between these risk factors and perceived disease risk.

**Conclusion:**

Although participants seemed to have some understanding of disease causation, they only partially translated their risk factors into accurate perceptions of risk. To improve understanding of risk information, health professionals may need to educate patients on how personal risk factors can contribute to the development of diabetes and CVD.

## Introduction

Epidemiological models indicate that several factors contribute to the development of diabetes and cardiovascular disease (CVD). Many patients who develop diabetes or CVD have a history of physiological characteristics associated with a disordered metabolism such as insulin resistance, cholesterol abnormalities, hypertension, and abdominal obesity (1,2). These metabolic abnormalities increase with age and are mostly caused by a combination of genetic and behavioral factors (ie, unhealthy eating habits, physical inactivity, and smoking). Reflecting the consequences of interactions among multiple genetic and behavioral factors, a family disease history is a strong and independent risk factor for diabetes (3,4) and CVD (5-7), and having diabetes increases the risk of CVD by 2 to 4 times (2).

Some researchers have argued that perceptions of disease risk are based on matching personal risk factor information with beliefs about risk factors and causes of disease (8-11). If a risk factor does not fit within beliefs about disease causation, it is unlikely to increase perceived risk. For example, smoking may only be associated with higher risk of a disease if it is considered an important cause of the disease.

The few studies on causal beliefs for diabetes and CVD show that most people believe that both lifestyle factors and genetic factors could cause these diseases (12-14). However, these studies did not examine the role of causal beliefs in perceived disease risk or make comparisons between diseases. To improve the effectiveness of disease risk communication, a better understanding of how people perceive their risk of developing a disease is needed.

The objective of this study was to examine the extent to which causal beliefs about diabetes and CVD affected the relationship between self-reported risk factors and perceived disease risk. We examined how people perceive their risk of diabetes and CVD in relation to their actual risk according to epidemiological models.

## Methods

### Design, sample, and procedure

We used data from a cross-sectional study performed in 2007. The sample was recruited from a database of a population-based screening program to identify people with undiagnosed diabetes among inhabitants of a semirural region of the Netherlands, carried out from 1998 through 2000 (15). Participants in this program first filled out a Symptom Risk Questionnaire, which included questions about age, family history of diabetes, and body mass index (BMI). Participants who screened positive (scores ≥6) were subsequently biochemically tested for diabetes. High scores indicated a high risk for diabetes and CVD (16). We randomly selected a subset (n = 319) of all people who screened positive and who did not have diabetes at the time of testing and mailed them a letter, describing this study. The letters included study information and informed consent forms. Participants who reported not having diabetes and who signed and returned the informed consent form were sent a postal questionnaire (participation rate was 80%, n = 255). The VU University Medical Center ethical committee in Amsterdam approved the study protocol. Participants provided data on age, sex, marital status, and educational level.

### Measures

**Coding of self-reported risk factors.** We used 4 measures to assess physiological risk factors associated with a disordered metabolism. Participants were asked if they had been diagnosed with high cholesterol or blood pressure. For both items, we coded "yes" responses as 1 and "no" responses as 0. With self-reported weight (in kg) and height (in m), we calculated BMI. Normal weight (BMI <25.0 kg/m^2^) was coded as 0, overweight (BMI 25.0-29.9 kg/m^2^) as 1, and obese (BMI ≥30.0 kg/m^2^) as 2.

We collected data on 3 behavioral risk factors: 1) unhealthy diet and eating habits, 2) lack of physical activity, and 3) smoking. The first 2 behaviors were assessed by the extent of agreement with the statements, "I attend to my diet and see to it that I eat healthy every day" and "I see to it that I am sufficiently physically active every day" (explained in the instructions to participants as eating food with little saturated fat and at least 2 pieces of fruit and 200 g of vegetables every day and at least half an hour of moderately intense physical activity, such as walking, biking, swimming or gardening, 5 d/wk); responses ranged from 1 (completely disagree) to 5 (completely agree). For both statements, responses were dichotomized: 1, 2, and 3 were recoded as 1, and categories 4 and 5 were coded as 0. Participants also indicated which category best described their smoking behavior (response categories: "I am a smoker," "I stopped smoking less than 2 years ago," "I stopped smoking more than 2 years ago," and "I have never smoked [with any regularity])." Nonsmokers and ex-smokers were assigned a score of 0, and current smokers were assigned a score of 1*.* Participants also provided information on how many (0, 1, or ≥2) and which of their 1st-degree family members (parents and siblings) were affected by diabetes and CVD, respectively.

**Perceived disease risk.** With 2 items for diabetes and CVD, we assessed perceived disease risk: "How likely do you think it is that you will get diabetes [or CVD] within the next 10 years?" (1 [very unlikely] to 7 [very likely]), "Based on your feelings, what is the chance that you will develop diabetes [or CVD] within the next 10 years?" (1 [very low] to 7 [very high]). Reliability (Spearman-Brown coefficient) of this measure was *r*_sh_ =. 93 for both diabetes and CVD.

**Causal beliefs.** In a list of possible causes of diabetes (or CVD), taken from the revised Illness Perception Questionnaire (17), participants indicated for each cause the extent to which they believed it could be a cause of diabetes (or CVD). For this present study, we selected the causes associated with established risk factors: "advancing age," "unhealthy diet or eating habits," "lack of physical activity," "smoking," and "hereditary, diabetes [CVD] runs in the family." In addition, participants indicated the extent to which they believed "being overweight" could be a cause of diabetes and the extent to which they believed "raised cholesterol levels," "raised blood pressure," and "having diabetes" could increase the risk of CVD. Responses for all items were assessed on 5-point rating scales from 1 (definitely not) to 5 (definitely).

### Statistical analyses

Data analyses were performed by using SPSS 15.0 (SPSS, Inc, Chicago, Illinois). To describe the characteristics of the sample and perceived disease risk and causal beliefs for diabetes and CVD, we generated means, standard deviations, and frequencies. Cases with missing values were deleted pairwise from the subsequent analyses. Simple and multiple regression analyses were used to examine the predictive value of the 9 risk factors on perceived risk of diabetes and CVD. To explore the moderating role of causal beliefs on perceptions of disease risk, we performed a series of separate hierarchical multiple regression analyses for each risk factor. In the first step, we entered the risk factor and the associated causal belief in the regression; in the second step, we added an interaction term (product of risk factor and causal belief) to the regression. A moderator effect is present if the interaction term explains a significant amount of variance in perceived risk (P <.05). To compare across diseases, we performed paired-samples *t *tests to assess the difference between perceived risk of diabetes and of CVD and differences in causal beliefs.

## Results

The mean age of participants was 66 years (standard deviation [SD] 5, range 57-79). Most participants were either married or living together, education level was generally low, and a high percentage of participants were overweight (Table 1).

### Perceived disease risk and predictive value of risk factors

Mean scores of both measures of perceived risk measures were below the scale midpoint (Table 2). Mean scores for perceived risk of diabetes were significantly lower than means scores for perceived risk of CVD.

A positive family history of diabetes and lack of physical activity were positively associated with perceived diabetes risk, and being 65 years or older was negatively associated with perceived diabetes risk (Table 2). Raised cholesterol levels, family history of CVD, unhealthy diet and eating habits, and lack of physical activity were positively associated with perceived CVD risk; being 65 years or older and having a family history of diabetes were negatively associated with perceived CVD risk. Taken together, self-reported risk factors accounted for 11% of the variance in perceived risk of diabetes and 18% of the variance in perceived risk of CVD (Table 2).

### Causal beliefs and the relationship between risk factors and perceived disease risk

Causal beliefs concerning behavioral factors (unhealthy diet, lack of physical activity, and smoking) were weaker for diabetes than for CVD (Figure). Mean scores for causal beliefs about diabetes ranged from 2.9 (SD, 1.2) for "smoking" to 4.0 (SD, 1.0) for "being overweight" (Figure). Mean scores for beliefs about CVD were at least ≥4.0, except for "advancing age" (mean, 3.7; SD, 0.9) and "having diabetes" (mean, 3.4; SD, 1.1).

**Figure 1.**
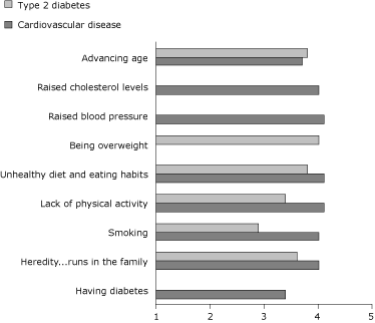
Causal beliefs for diabetes and cardiovascular disease, the Netherlands, 2007 (n = 255). Participants indicated on a scale of 1 to 5 for each cause the extent to which they believed it could be a cause (or risk factor) of diabetes or cardiovascular disease.

**Table:** 

**Causes and risk factors**	**Diabetes, Mean (SD)**	**Cardiovascular Disease, Mean (SD)**	**Difference**	
			***t***	***P* value**
Advanced age	3.8 (1.0)	3.7 (0.9)	*t*_232_ = − 1.07	<.001
Raised cholesterol levels	NA	4.0 (1.0)	NC	NC
Raised blood pressure	NA	4.1 (1.0)	NC	NC
Being overweight	4.0 (1.0)	NA	NC	NC
Unhealthy diet or eating habits	3.8 (1.2)	4.1 (1.1)	*t*_237_ = 3.94	<.001
Lack of physical activity	3.4 (1.2)	4.1 (1.0)	*t*_238_ = 7.93	<.001
Current smoker	2.9 (1.2)	4.0 (1.2)	*t*_235_ = 13.17	<.001
Heredity . . . runs in the family	3.6 (1.3)	4.0 (1.1)	*t*_236_ = 3.77	<.001
Having diabetes	NA	3.4 (1.1)	NC	NC

Abbreviations: SD, standard deviation; NC, not calculated NA, not assessed.

For diabetes, we found significant interaction effects between risk factors and causal beliefs for advancing age and being overweight (Table 3a). For advanced age, explained variance in perceived risk of diabetes increased from 3% for age category alone to 6% when the interaction term, the risk factor causal belief, was added; for being overweight, explained variance in perceived risk of diabetes increased from 0% to 3%. For CVD, we found significant risk factor causal belief interaction effects for smoking and heredity (Table 3b). For smoking, explained variance in perceived risk of CVD increased from 0% to 3% and for family history, explained variance increased from 3% to 8%. Other causal beliefs for diabetes and CVD did not significantly moderate the relationship between risk factor and perceived disease risk.

## Discussion

Participants in this sample of older adults at risk for CVD and diabetes only partially translated self-reported risk factor information into perceptions of risk. They seemed to have a somewhat better understanding of the association between risk factors and CVD risk than between risk factors and diabetes risk. Although for some risk factors, causal beliefs strengthened the association between self-reported risk factor and perceived disease risk (ie, for perceived risk of diabetes, association between advancing age and being overweight, and for perceived risk of CVD, association between smoking and heredity), for other risk factors, no moderator effects of causal beliefs were found.

These results support earlier findings that family disease history is associated with perceived risk of diabetes (18-21) and CVD (22-24). We found that believing more strongly that heredity could be a cause of disease strengthened the association between family history of CVD and perceived CVD risk but not between family history of diabetes and perceived diabetes risk. It is possible that the association between family history and perceived risk of diabetes is best explained by the salience of diabetes in memory, which increases with the number of and closeness to people they know with diabetes, rather than family history and heritability of diabetes per se (13). There was an unexpected negative association between family history of diabetes and perceived CVD risk. Any explanation is highly speculative and further studies are needed to determine the exact relationship between family history of diabetes and perceptions of CVD risk.

Similar to other studies conducted in the Netherlands (20,24) and the United States (18,21), we found that people older than 65 years had lower perceptions of risk than younger people. Considering that this age group has a higher objective risk of diabetes (17) and CVD (25), their lower risk perceptions may be viewed as misconceptions. In addition, the negative relationship between age and perceived diabetes risk was even more pronounced in participants who viewed advancing age as an important cause of diabetes. One explanation is that after a certain age, people may think that if they were likely to develop a disease, they would already have developed the disease. People may also minimize their risk as a defensive mechanism.

In general, associations between risk factors and perceived disease risk were weak. Confirming the findings of previous studies (20,21), we found that neither being overweight nor smoking were associated with higher perceived risk of diabetes. Contrary to previous findings, we also found that perceived risk of CVD was not primarily associated with being a smoker (24,26). However, most participants indicated that they believed being overweight could cause diabetes, and smoking could cause CVD. One explanation for this apparent paradox may be that in general, people show an optimistic bias when considering their own health risk (27). People believe that they are healthier than others not because they have inaccurate knowledge about the causes of disease but because they do not correctly apply this knowledge to themselves. In particular, they may believe their actions contributing to risk (eg, smoking) are outweighed by their actions to prevent risk (eg, engaging in sufficient physical activity). Notably, being overweight and believing that overweight could be a cause of diabetes was associated with higher perceptions of diabetes risk. Similarly, being a smoker and believing that smoking could be a cause of CVD was associated with higher perceptions of CVD risk.

For other risk factors, causal beliefs did not strengthen the associations between risk factor and perceived risk. One explanation may be that causal beliefs were assessed with closed questions, which tend to overestimate actual knowledge of disease causation (12). The absence of a moderator effect for causal beliefs concerning unhealthy diet and eating habits and lack of physical activity may also be explained by the phrasing of the items assessing these factors. Participants were not asked to report on their behavior as such, but to provide qualitative judgments. Responses included, "I attend to my diet and see to it that I eat healthy every day," and "I see to it that I am sufficiently physically active every day." Disagreement with these items already indicates some awareness of a causal link between self-reported behavior and health risk.

Previous studies comparing perceived risk of diabetes and CVD show inconsistent results. Although DiLorenzo et al (28) found that people perceive their risk of developing CVD as higher than the risk of developing diabetes, Wang et al (29) found no difference in perceived risk between diabetes and CVD. In our study, we found that participants perceived their risk for CVD as higher than for diabetes. Furthermore, self-reported risk factors explained more variance in perceived CVD risk than in perceived diabetes risk. Participants also demonstrated a better knowledge about the causes of CVD risk than about the causes of diabetes risk. The general public is probably better informed about risk factors and causes for CVD because it is the leading cause of death in the Netherlands and is more prevalent than diabetes. Notably, having diabetes was not generally understood as a risk factor for CVD. Other studies also show that even among people diagnosed with diabetes, the link between having diabetes and higher risk of CVD is not clearly understood (24,26).

This study has some limitations. First, it presents cross-sectional data and therefore, does not show the dynamic relationship between risk factors, perceived disease risk, and causal beliefs. If a personal risk factor is seen as increasing disease risk or as an important cause of disease, then a person may be motivated to engage in preventive behavior, which in turn may reduce the number of risk factors. Second, people may not accurately report on risk factors. Third, the representativeness of the study population is unknown. The sample participated in a population-based diabetes screening program some years earlier; therefore, their understanding of diabetes risk might have been better than that of the average at-risk population. On the other hand, being tested for diabetes and not having diabetes could have minimized the belief that the risk factors had any personal relevance and might have reduced the moderating effect of causal beliefs.

Our results confirm that people's understanding of their risk of developing diabetes and CVD is limited. In general, people have some knowledge of disease causation, in particular of CVD, but only partially use this knowledge to link personal risk factors to an increased risk. To address misconceptions and improve understanding of risk information, health professionals may need to educate patients on the causes of disease and how personal risk factors can contribute to the development of diabetes and CVD.

## Figures and Tables

**Table 1:** Self-reported Characteristics of Participants (n = 255) who Reported Perceived Disease Risk, the Netherlands, 2007

**Characteristic**	n (%)
**Demographic variables**	
Age, mean (SD), y	66 (5)
≥65 y	156 (61)
Male	132 (52)
Married/living together	200 (78)
**Education^a^**	
Low	140 (55)
Medium	71 (28)
High	35 (14)
**Risk factors**	
**Physiological risk factors**	
Raised cholesterol levels	78 (31)
Raised blood pressure	125 (49)
Overweight (BMI ≥25 kg/m^2^)	201 (79)
Obese (BMI ≥30 kg/m^2^) (% of overweight)^b^	88 (44)
**Behavioral risk factors**	
Unhealthy diet or eating habits^c^	84 (33)
Lack of physical activity^d^	97 (38)
Current smoker	35 (14)
**Family disease history^e^**	
Diabetes	112 (44)
CVD	149 (58)
Both diabetes and CVD	81 (32)

Abbreviations: SD, standard deviation; BMI, body mass index; CVD, cardiovascular disease.

a Low: primary school, lower level of secondary school, or lower vocational training. Medium: higher level of secondary school, or intermediate vocational training. High: higher vocational training or university.

b Of the 201 (79%) participants who were overweight, 88 (44%) were obese.

c Lack of agreement with the statement, "I attend to my diet and see to it that I eat healthy every day."

d Lack of agreement with the statement, "I see to it that I am sufficiently physically active every day."

e At least one 1st-degree relative with diabetes or CVD.

**Table 2:** Association of Perceived Risk of Type 2 Diabetes and Cardiovascular Disease (CVD) with Risk Factors, the Netherlands, 2007

Characteristic	Perceived Risk of Diabetes				Perceived Risk of CVD			

**Simple regression analyses** a								

**Risk factors**	β	*t*	R^2^	*P* Value	β	*t*	R^2^	*P* Value
Advanced age^b^	−.17	*t*_239_ = −2.61	.03	.01	−.13	*t*_236_ = −1.98	.02	.05
Raised cholesterol levels	.08	*t*_235_ = 1.25	.01	.21	.28	*t*_233_ = 4.36	.08	<.001
Raised blood pressure	.06	*t*_235_ = .96	0	.34	.10	*t*_233_ = 1.48	.01	.14
BMI^c^	.05	*t*_230_ = .62	0	.50	.05	*t*_228_ = .72	0	.50
Unhealthy diet/eating habits^d^	.11	*t*_240_ = 1.67	.01	.10	.13	*t*_238_ = 2.07	.02	.004
Lack of physical activity^e^	.16	*t*_240_ = 2.50	.03	.01	.16	*t*_238_ = 2.54	.03	.01
Current smoker	−.05	*t*_235_ = −.71	0	.48	.05	*t*_232_ = −.75	0	.46
Family history diabetes^b^	.22	*t*_233_ = 3.48	.05	.001	−.18	*t*_231_ = −2.48	.03	.007
Family history CVD^b^	.02	*t*_238_ =.39	.00	.70	18	*t*_236_ = 2.82	.03	.005

**Multiple regression analyses^f^**								

**Risk factors**	**β**	***t*_222_**	**R^2^**	***P* Value**	**β**	***t*_220_**	**R^2^**	***P* Value**

Advanced age^b^	−.13	−1.95	.11	.05	−.14	−2.16	.18	.03
Raised cholesterol levels	.06	.92		.36	.23	3.61		<.001
Raised blood pressure	.05	.78		.44	.03	.40		.70
BMI^c^	.01	.12		.91	.02	.36		.72
Unhealthy diet/eating habits^d^	.07	1.08		.28	.02	.29		.77
Lack of physical activity^e^	.11	1.72		.08	.11	1.75		.08
Current smoker	−.04	−.60		.55	.03	.45		.66
Family history of diabetes^g^	.23	3.53		<.001	−.20	−3.22		.001
Family history CVD^g^	−.01	−.09		.93	.21	3.32		.001

Abbreviations: SD, standard deviation; BMI, body mass index.

a Mean (SD) range 1-7. For perceived risk of diabetes, 2.9 (1.4) and for perceived risk of CVD, 3.8 (1.6). Difference, *P* value for perceived risk of CVD, *t*_235_ = 7.97, <.001.

b Age in years: <64 = 0; ≥65 = 1.

c BMI <25.0 kg/m^2^ = 0, 25.0 kg/m^2^-29.9 kg/m^2^ = 1, ≥30.0 kg/m^2^ = 2.

d Lack of agreement with the statement, "I attend to my diet and see to it that I eat healthy every day."

e Lack of agreement with the statement, "I see to it that I am sufficiently physically active every day."

f Explained variance of all risk factors for multiple regression analysis: for diabetes, F_9,222_ = 3.03, *P* = .002; for CVD, F_9,220_ = 5.42, *P* < .001.

g Number of affected 1st-degree relatives: 0, 1, or ≥2.

**Table 3a.** Hierarchical Multiple Regression Analyses for Perceived Risk of Diabetes on Risk Factors, the Netherlands, 2007^a^

Causes and Risk Factors	1st Step			2nd Step		

	*ß*	*P* value	R^2^	*ß*	*P* Value	R^2^
**Advanced age^b^**	.17	.01	.04	.34	.19	.06
Causal belief	.11	.10		.28	.01	
Interaction				.55	.04	
**BMI ≥25 kg/m^2c^**	.04	.54	0	.63	.03	.03
Causal belief	.04	.60		.18	.11	
Interaction				.75	.02	
**Unhealthy diet/eating habits^d^**	.11	.10	.01	.13	.56	.02
Causal belief	.03	.64		.03	.76	
Interaction				.25	.26	
**Lack of physical activity^e^**	.15	.02	.03	.24	.26	.03
Causal belief	.06	.32		.08	.29	
Interaction				.09	.68	
**Current smoker^f^**	.04	.52	0	.27	.13	.01
Causal belief	.05	.50		.01	.84	
Interaction				.25	.16	
**Family history of diabetes^g^**	.20	.002	.06	.15	.55	.06
Causal belief	.09	.16		.08	.30	
Interaction				.06	.82	

a The extent to which participants believed this could be a cause (or risk factor) of diabetes.

b Age in years, <64 = 0; ≥65 = 1.

c BMI <25.0 kg/m^2^ = 0, 25.0 kg/m^2^-29.9 kg/m^2^ = 1, ≥30.0 kg/m^2^ = 2.

d Lack of agreement with the statement, "I attend to my diet and see to it that I eat healthy every day."

e Lack of agreement with the statement, "I see to it that I am sufficiently physical active every day."

f Participants who identified themselves as being a smoker.

g Number of affected 1st-degree relatives: 0, 1, or ≥2.

**Table 3b.** Hierarchical Multiple Regression Analyses for Perceived Risk of Cardiovascular Disease on Risk Factors, the Netherlands, 2007^a^

Causes and Risk Factors	1st Step			2nd Step		

	*ß*	*P* Value	R^2^	*ß*	*P* Value	R^2^
**Advanced age^b^**	.12	.07	.03	.52	.09	.03
Causal belief	.10	.13		.04	.76	
Interaction				.42	.17	
**Raised cholesterol levels**	.26	<.001	.08	.35	.32	.09
Causal belief	.05	.41		0	.98	
Interaction				.63	.08	
**Raised blood pressure**	.08	.24	.03	.18	.57	.03
Causal belief	.13	.06		.09	.28	
Interaction					.41	
**Unhealthy diet/eating habits^c^**	.13	.04	.02	.35	.25	.03
Causal belief	.01	.84		.04	.55	
Interaction				.50	.10	
**Lack of physical activity^d^**	.16	.01	.03	.29	.29	.03
Causal belief	.03	.68		.01	.92	
Interaction				.13	.64	
**Current smoker^e^**	.05	.52	0	.56	.06	.03
Causal belief	.02	.50		.03	.68	
Interaction				.63	.02	
**Family history of CVD^f^**	.16	.02	.04	.90	.005	.08
Causal belief	.06	.38		.12	.18	
Interaction				1.16	.001	

a The extent to which participants believed this could be a cause (or risk factor) of cardiovascular disease.

b Age in years: <64 = 0; ≥65 = 1.

c Lack of agreement with the statement, "I attend to my diet and see to it that I eat healthy every day."

d Lack of agreement with the statement, "I see to it that I am sufficiently physical active everyday."

e Participants who identified themselves as being a smoker.

f Number of affected 1st-degree relatives: 0, 1, ≥ 2.

## References

[1] D'Agostino RB, Hamman RF, Karter AJ, Mykkanen L, Wagenknecht LE, Haffner SM (2004). Cardiovascular disease risk factors predict the development of type 2 diabetes. Diabetes Care.

[2] Smith SC (2007). Multiple Risk Factors for Cardiovascular Disease and Diabetes Mellitus. Am J Med.

[3] Harrison TA, Hindorff LA, Kim H, Wines RC, Bowen DJ, McGrath BB (2003). Family history of diabetes as a potential public health tool. Am J Prev Med.

[4] Valdez R, Yoon PW, Liu T, Khoury MJ (2007). Family history and prevalence of diabetes in the U.S. population: the 6-year results from the National Health and Nutrition Examination Survey (1999-2004). Diabetes Care.

[5] Kardia SL, Modell SM, Peyser PA (2003). Family-centered approaches to understanding and preventing coronary heart disease. Am J Prev Med.

[6] Nasir K, Michos ED, Rumberger JA, Braunstein JB, Post WS, Budoff MJ (2004). Coronary artery calcification and family history of premature coronary heart disease sibling history is more strongly associated than parental history. Circulation.

[7] Murabito JM, Pencina MJ, Nam BH, D'Agostino RB, Wang TJ, Lloyd-Jones D (2005). Sibling cardiovascular disease as a risk factor for cardiovascular disease in middle-aged adults. JAMA.

[8] Leventhal H, Kelly K, Leventhal EA (1999). Population risk, actual risk, perceived risk, and cancer control: a discussion. J Natl Cancer Inst Monogr.

[9] Weinstein N (1999). What does it mean to understand risk? Evaluating risk comprehension. J Natl Cancer Inst Monogr.

[10] Cameron L Conceptualizing and assessing risk perceptions: a self-regulatory perspective. Paper presented at the National Cancer Institute workshop on conceptualizing and measuring risk perception.

[11] Gerend MA, Aiken LS, West SG, Erchull MJ (2004). Beyond medical risk: investigating the psychological factors underlying women's perceptions of susceptibility to breast cancer, heart disease, and osteoporosis. Health Psychol.

[12] Sanderson SC, Waller J, Jarvis MJ, Humphries SE, Wardle J (2009). Awareness of lifestyle risk factors for cancer and heart disease among adults in the UK. Patient Educ Couns.

[13] Walter FM, Emery J, Braithwaite D (2004). Lay understanding of familial risk of common chronic diseases: a systematic review and synthesis of qualitative research. Ann Fam Med.

[14] Pijl M, Henneman L, Claassen L, Detmar SB, Nijpels G, Timmermans DRM (2009). Family history of diabetes: exploring perceptions of people at risk in the Netherlands. Prev Chronic Dis.

[15] Spijkerman AMW, Adriaanse MC, Dekker JM, Nijpels G, Stehouwer CDA, Bouter LM (2002). Diabetic patients detected by population-based stepwise screening already have a diabetic cardiovascular-risk profile. Diabetes Care.

[16] Ruige JB, de Neeling JN, Kostense PJ, Bouter LM, Heine RJ (1997). Performance of an NIDDM screening questionnaire based on symptoms and risk factors. Diabetes Care.

[17] Moss-Morris R, Weinman J, Petrie KJ, Horne R, Cameron L, Buick D (2002). The Revised Illness Perception Questionnaire (IPQ-R). Psychol Health.

[18] Harwell TS, Dettori N, Flook BN, Priest L, Williamson DF, Helgerson SD (2001). Preventing type 2 diabetes: perceptions about risk and prevention in a population-based sample of adults > or = 45 years of age. Diabetes Care.

[19] Hariri S, Yoon PW, Qureshi N, Valdez R, Scheuner MT, Khoury MJ (2006). Family history of type 2 diabetes: a population-based screening tool for prevention?. Genet Med.

[20] Adriaanse MC, Twisk JWR, Dekker JM, Spijkerman AM, Nijpels G, Heine RJ, Snoek FJ (2008). Perceptions of risk in adults with a low or high risk profile of developing type 2 diabetes; a cross-sectional population-based study. Patient Educ Couns.

[21] Gallivan J, Brown C, Greenberg R, Clark CM (2009). Predictors of Perceived Risk of the Development of Diabetes. Diabetes Spectr.

[22] Hunt K, Davison C, Emslie C, Ford G (2000). Are perceptions of a family history of heart disease related to health­related attitudes and behaviour?. Health Educ Res.

[23] Montgomery GH, Erblich J, DiLorenzo T, Bovbjerg DH (2003). Family and friends with disease: their impact on perceived risk. Prev Med.

[24] Lobo CM, Keus IM, Jenks KM, Akkermans RP, Hulscher ME (2004). Perceptions of cardiovascular risk among patients with hypertension or diabetes. Patient Educ Couns.

[25] Conroy RM, Fitzgerald AP, Sans S, De Backer G (2003). Estimation of ten-year risk of fatal cardiovascular disease in Europe: the SCORE project. Eur Heart J.

[26] van der Weijden T, van Steenkiste B, Stoffers HE, Timmermans DR, Grol R (2007). Primary prevention of cardiovascular diseases in general practice. Mismatch between cardiovascular risk and the patient's risk perception. Med Decis Making.

[27] Weinstein ND (1987). Unrealistic optimism about susceptibility to health problems: conclusions from a community-wide sample. J Behav Med.

[28] DiLorenzo TA, Schnur J, Montgomery GH, Erblich J, Winkel G, Bovbjerg DH (2006). A model of disease-specific worry in heritable disease: the influence of family history, perceived risk and worry about other illnesses. J Behav Med.

[29] Wang C, O'Neill SM, Rothrock N, Gramling R, Sen A, Acheson LS (2008). Comparison of risk perceptions and beliefs across common chronic diseases. Prev Med.

